# Detection of equine parapoxvirus in the stable environment during pastern dermatitis outbreak in Finland

**DOI:** 10.1186/s13028-026-00870-9

**Published:** 2026-06-30

**Authors:** Sanna Tervo, Kirsi Aaltonen, Katja Hautala, Mira Utriainen, Luukas Pennström, Tarja Sironen, Paula M. Kinnunen, Jenni Pettersson

**Affiliations:** 1 Department of Veterinary Biosciences, Faculty of Veterinary Medicine, University of Helsinki, Agnes Sjöbergin Katu 2, 00790 Helsinki, Finland; 2 Department of Virology, Faculty of Medicine, University of Helsinki, Haartmaninkatu 3, 00290 Helsinki, Finland; 3 Institute of Biomedicine, Faculty of Medicine, University of Turku and University Hospital of Turku, Kiinamyllynkatu 10, 20520 Turku, Finland

**Keywords:** Environmental sampling, Equine parapoxvirus, Diagnostics, Pastern dermatitis, PCR, Virus transmission

## Abstract

**Background:**

Equine parapoxvirus (EqPPV) has emerged as a cause of recurring pastern dermatitis outbreaks in horses in Finland. First detected in 2013, EqPPV has since been associated with potentially hundreds of clinical cases and suspected zoonotic transmission. Based on serological data and field reports, EqPPV causes pastern dermatitis outbreaks in Finland every few years. This study aimed to investigate the environmental presence and plausible transmission dynamics of the virus to prepare for future outbreaks and give targeted guidelines to stable staff and veterinarians.

**Results:**

After a large-scale EqPPV outbreak affecting hundreds of horses occurred in Finland in winter 2021–2022, PCR diagnostics was carried out whenever EqPPV was suspected to monitor the situation (25 trotting horses from 20 stables between April 2022 and May 2025). Following a period with no detected EqPPV cases from April 2022 to January 2025, a new outbreak occurred in early 2025. Between February and May 2025, the majority of Finnish trotting stables (8/10) submitting diagnostic equine samples, tested positive for EqPPV DNA. Environmental samples (n = 161) were collected from six case stables and two control stables from various surfaces in direct or indirect contact with horses. EqPPV DNA was detected on grooming tools, stall infrastructure, bedding, materials handled by humans, and hands of humans, suggesting multiple possible indirect transmission routes and large-scale spread of EqPPV DNA in infected stables. Notably, 83.3% (5/6) of the tested case stables had EqPPV PCR-positive environmental samples and 17.4% (24/138) of all environmental samples tested positive.

**Conclusions:**

This study confirms a recent EqPPV outbreak in Finnish trotting stables and demonstrates the presence of EqPPV DNA in clinically affected horses and the stable environment, e.g. common spaces and shared equipment. Detection of EqPPV DNA on common equipment and spaces, and on personnel shows a widespread environmental distribution during the outbreak. Overall, these findings underscore the high potential for reoccurring outbreaks and the importance of continued surveillance and early implementation of hygiene and preventative practices to minimize the effect of future outbreaks.

**Supplementary Information:**

The online version contains supplementary material available at https://doi.org/10.1186/s13028-026-00870-9.

## Background

Poxviruses (family *Poxviridae*) are large, enveloped viruses with a 128–375 kbp double-stranded DNA genome. They are divided into two subfamilies: *Entomopoxvirinae* infecting insects and *Chordopoxvirinae* infecting vertebrates and are important pathogens of humans, livestock, and wildlife [1]. Several poxviruses are zoonotic and capable of transmitting between animals and humans.

The genus *Parapoxvirus* (PPV) comprises several viruses known to infect domestic animals and wildlife, including bovine papular stomatitis virus (BPSV), orf virus (ORFV), pseudocowpox virus (PCPV), parapoxvirus red deer (RDPV), seal parapoxvirus (GSEPV), and equine parapoxvirus (EqPPV). Several PPVs, including ORFV and PCPV, are zoonotic, whereas zoonotic infections by EqPPV have not been confirmed [2–5]. These viruses generally cause localized skin lesions [1]. In Finland, different parapoxviruses have been detected in various domestic and wild animals: ORFV in sheep and reindeer, BPSV in cattle, PCPV in reindeer, and EqPPV in horses [4, 6–9]. Human infections with ORFV, PCPV, and BSPV can occur through contact with infected animals [2, 3, 5, 10]. For example, PPV infections have been reported in humans in Europe through contact with cows (BSPV) or through handling raw animal intestinal organs (ORFV) [10].

EqPPV was first identified in a sick horse in Finland in 2013 [4]. Around the same time, two cases were reported in the United States [3] involving women infected with a poxvirus that shared 99–100% nucleotide identity with EqPPV detected in Finland, based on a 585 bp sequence (KR827441) fragment. One was immunocompromised and had been in contact with animals including horses and poultry; the other had sustained a rope burn after handling donkeys in Tanzania. Both developed lesions consistent with poxvirus infection [3]. In winter 2021–2022, EqPPV caused a large outbreak of pastern dermatitis in the Finnish trotter population, affecting potentially hundreds of horses. On average, one-third of horses in infected stables were affected. Many developed long-lasting clinical signs and had to be taken out of training, resulting in economic losses. Field reports described pastern dermatitis outbreaks with similar clinical signs in wintertime 2015 and 2019 [11, 12]. Suspected cases have also been reported in Sweden and the Netherlands (personal communication with local veterinarians), and EqPPV was recently confirmed by a laboratory in Sweden [13]. EqPPV has been shown to cause a rapid immune response that can last at least a year, however, the length of immunity and level of protection remain unknown [12]. Antibodies against other PPVs, such as ORFV and BPSV, appear to provide only limited protective immunity as they do not prevent reinfection [14, 15].

In the absence of treatment or vaccination, interrupting transmission remains a key objective in controlling pastern dermatitis. However, this is complicated by the limited understanding of EqPPV transmission dynamics, and therefore current recommendations are largely extrapolated from studies on other PPVs such as ORFV and BPSV. For example, ORFV is known to persist in the environment and remain infectious in dried scabs and in contaminated farm materials for prolonged periods, facilitating indirect transmission and persistence between outbreaks [16, 17]. Detection of ORFV and BPSV DNA in farm environments supports the possibility of their indirect transmission via contaminated surfaces, raising the hypothesis that EqPPV may similarly persist in stable environments and contribute to transmission between horses [18–20]. Understanding how widespread EqPPV is in a stable environment, e.g. common spaces and shared equipment, is needed to provide insight into potential transmission pathways between individuals. This knowledge would support the development of more targeted preventative measures and guidelines for managing infected stables.

The number of reports from stable staff and veterinarians describing horses showing clinical signs of pastern dermatitis, consistent with EqPPV infection, increased again at the beginning of February 2025, three years after the previous outbreak. The aim of this study was to investigate the transmission of EqPPV by testing environmental samples from stables affected by pastern dermatitis. Additionally, we report the results of EqPPV diagnostics of pastern dermatitis cases carried out since the winter 2021–2022 outbreak to increase understanding of outbreak dynamics.

## Methods

### Disease diagnostics

Following the winter 2021–2022 outbreak, the EqPPV situation was monitored through ongoing communication with veterinarians and the racing community via social media, targeted press releases, and conferences and by conducting PCR diagnostics on samples from horses showing clinical signs of pastern dermatitis, consistent with EqPPV (n = 25; April 2022—May 2025) (Additional File 1). Diagnostics were carried out from skin swabs with a specific, probe-based PCR validated and described earlier [11]. Analytic sensitivity of the PCR had been tested to be 10 viral genome copies/reaction. All samples sent for diagnostics were from Finland. The first day of noticing clinical signs, sampling day, type of clinical signs, number of affected horses, location of the stable, suspected infection source, and potential skin symptoms in humans were queried in the referral form. Samples were collected during routine veterinary diagnostics with the consent of horse owners. No ethical approval was required in compliance with EU directive 2010/63/EU and national act 497/2013. If affected horses were confirmed EqPPV-positive by PCR, willingness of the stable to participate in environmental sampling was ascertained.

### Collection of environmental samples

**Fig. 1:**
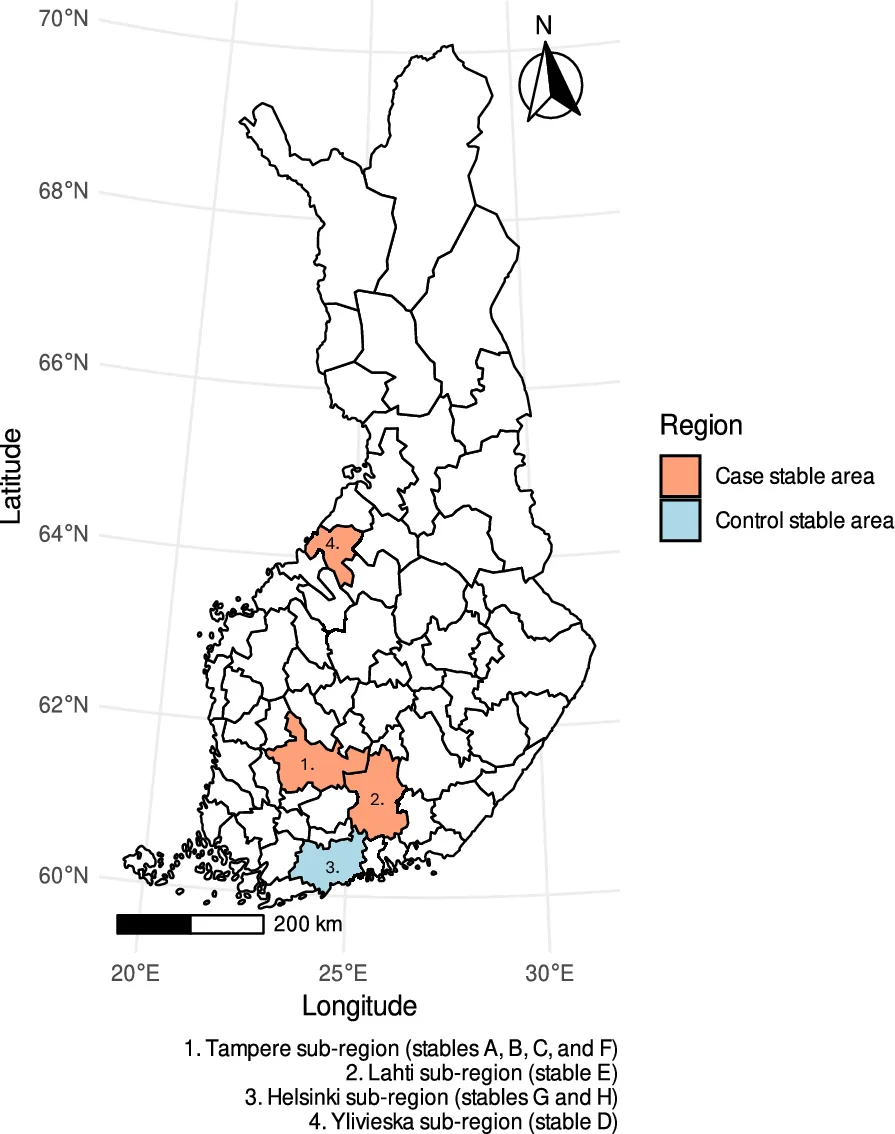
Collection regions of environmental samples in Finland. Case stables (A, B, C, D, E, and F) areas are presented in orange (Tampere sub-region, Lahti sub-region, Ylivieska sub-region) and control stables (G and H) are presented in blue (control area) (Helsinki sub-region). The figure was generated using R (version 4.5.0)

Environmental samples (n = 161) collected from eight Finnish stables during spring 2025 included six case stables (A-F) and two control stables (G and H); details are provided in Tables 1, 2, Fig. 1, and Additional File 1. Case stables were selected based on positive PCR results in diagnostics and had horses with clinical signs consistent with EqPPV infection during spring 2025. One control stable (G) had experienced EqPPV outbreak in 2022, confirmed by PCR earlier [11], but showed no pastern dermatitis cases in 2025, while another control stable (H) had no history of EqPPV. Environmental samples were collected with the permission of stable owners and staff.

**Table 1:** Information on stables included in environmental sample collection

Stable ID	Sub-region	Onset of clinical signs^a^	Environmental sampling date	N of affected horses^b^	Disease in 2021-2022^d^	Human symptoms^c,f^
A	Tampere	11 Jan 2025	5 Mar 2025	1/12 (1/1)	Yes	Yes
B^e^	Tampere	Early Mar 2025	7 Mar 2025	5/25 (1/1)	Yes	No
C^e^	Tampere	2 Mar 2025	7 Mar 2025	7/21 (1/1)	Yes	No
D	Ylivieska	4 Feb 2025	17 Mar 2025, 15–16 Jun 2025	12/17 (4/8)	Yes	Yes
E	Lahti	Early Mar 2025	18 Mar 2025, 6 Jun 2025	5/28 (3/3)	Yes	Yes
F	Tampere	25 Mar 2025	3 May 2025	1/4 (1/1)	Unclear	No
G	Helsinki	No disease in 2025	28 May 2025	0/19 (0/1)	Yes	NA
H	Helsinki	No disease in 2022 or 2025	12 Jun 2025	0/30 (0/3)	No	NA

^a^EqPPV confirmed in stables A-F by PCR prior to environmental sampling

^b^Based on reports off owners. The number of cases confirmed with PCR is in parentheses

^c^NA = Not applicable

^d^EqPPV confirmed in Stables D, E and G by PCR. For stables A, B, C, and F, the information is based on reports of typical clinical signs of pastern dermatitis

^e^Despite distance of tens of kilometers, some of the personnel were the same in stables B and C

^f^Answers to question: Did humans in contact with infected horses report skin symptoms in hands during the 2025 outbreak?

**Table 2:** Real-time PCR confirmed EqPPV samples presented by environmental categories

Stable ID	Proportion of positive samples	Category 1^a^	Category 2^a^	Category 3^a^	Category 4^a^
Case stables					
A	5.0% (2/40)	1/15	1/14	0/1	0/10
B	22.2% (2/9)	0/0	2/2	0/1	0/6
C	0.0% (0/6)	0/2	0/3	0/0	0/1
D	42.9% (12/28)	3/9	4/7	3/7	2/5
E	10.0% (5/50)	0/7	2/23	1/9	2/11
F	60.0% (3/5)	1/1	2/4	0/0	0/0
Total	17.4% (24/138)	14.7% (5/34)	20.8% (11/53)	22.2% (4/18)	12.1% (4/33)
Control stables					
G	0.0% (0/15)	0/2	0/5	0/4	0/3
H	0.0% (0/8)	0/1	0/5	0/1	0/1

^a^Categories: human contact points (1), animal contact points (2), shared equipment/places (3), and environmental surfaces (4)

Stables A-E had horses with clinical signs during the sampling. Stable F was sampled 2–3 weeks after the infected horse had recovered. Additionally, two affected horses in stables A and D were sampled again after 2 weeks and one month, respectively, from the initial sampling. Stables A-G were trotting stables, and stable H was a riding stable. The riding stable was chosen to be a negative control, as EqPPV circulation is considered more probable in trotting stables, while no PCR-confirmed EqPPV cases have been reported in riding stables. Samples were taken from boxes of both infected and neighboring horses, common spaces, individual and shared equipment, and personnel (sampler’s or handler’s gloves or shoes, wounds and scabs) (Additional File 2). Gloves and shoes were sampled after exiting the box of an affected horse, after which gloves were changed, and boots were cleaned to avoid cross-contamination. Samples were collected either with flocked swab (Puritan Medical Products, Guilford, ME, USA) or UTM tube with liquid transport medium (Copan, Brescia, Italy). Bedding, hay, and samples from muckheap were collected as such.

When possible, swab samples were taken from the affected horse/horses during environmental sampling, including both earlier PCR-confirmed and non-PCR-confirmed individuals (Additional File 3). Environmental samples were divided into four categories based on the sampling spot: human contact points (category 1), animal contact points (category 2), shared equipment/places (category 3), and environmental surfaces (category 4) (Table 2 and Additional File 2). Environmental samples from stables D and E were collected twice: once during the acute phase of the disease and again 2 months after the outbreak, targeting surfaces that had previously tested positive. The sample size varied between stables reflecting differences in stable size, accessibility, practices in the stables, time each stable was able to allocate for sampling, and number of available horses and environmental sampling sites at the time of collection, which influenced sample allocation across both stables and sample categories. Additionally, sampling was performed by a combination of researchers, stable owners, and veterinarians, which introduced some variability in sampling intensity. For this reason, the study was not powered to support formal statistical comparisons between groups.

After the outbreak, all case stables were asked how many horses had eventually developed clinical signs of pastern dermatitis, what preventative measures were taken to control the spread, did the stable have the disease during the previous outbreak in 2021–2022, and did humans develop symptoms in sites that were in contact with infected horses.

### Collection and handling of small wild mammal and bird samples

To study the potential animal reservoirs of EqPPV, samples were acquired from small mammals and birds in a racetrack (collected in 2024) that was heavily affected by EqPPV in winter 2021–2022. Two field mice (*Apodemus agrarius*) and three bank voles (*Myodes glareolus*) were provided by a professional pest control company (Anticimex) that had trapped rodents in the area. Additionally, faeces of birds (n = 4), rabbits (n = 6), big rodents (n = 2, exact species not determined), and small rodents/shrews (n = 4, exact species not determined) were collected next to the haystacks near the stable buildings. From the same locations, samples were also collected from wild rabbit urine-contaminated snow (n = 9) and combined into three pools. Rodents were dissected in the laboratory and hearts were collected into 500 µL of phosphate-buffered saline (PBS; like previously described [21] to detect orthopoxvirus antibodies in rodents). All samples were stored at -20 °C. Detailed information on the samples is provided in Additional File 4.

### Sample preparation and DNA extraction

Dry swabs from the environment and horses (n = 45) (Puritan) were incubated in 500 µL of sterile PBS for 15 min by vigorous vortexing every 5 min, followed by DNA extraction using the NucleoSpin Tissue kit (Macherey–Nagel, Düren, Germany) protocol “Support protocol for genomic DNA and viral DNA from blood samples” [22]. Copan swabs from the environment and horses (n = 123) were processed identically, omitting the PBS incubation step. Bedding (n = 12), hay (n = 2) and muckheap (n = 2) samples were combined with 25–40 mL PBS, incubated at room temperature (RT) for 1 h with shaking, and filtered sequentially through Whatman MN615 ¼ filter and 0.45 µm polyethersulfone filters. The filtrate was concentrated using Amicon Ultra-15 Centrifugal Filter Units (Merck, Darmstadt, Germany), then extracted using NucleoSpin protocol as above. Snow/urine samples (n = 9) were pooled into three groups (2–3 samples each), concentrated with Amicon Ultra-15 units, and extracted as above. Faecal samples from rabbits, rodents and birds were homogenized in 2 mL of NaCl, vortexed, and filtered through 0.45 µm polyethersulfone filters. DNA was extracted using the NucleoSpin protocol as above. All DNA samples were extracted according to the manufacturer’s instructions, except samples were eluted in 50 µL of elution buffer instead of 100 µL and stored at -20 °C until further analysis. Negative extraction control (water) was used to detect potential contamination during DNA extraction.

### Detection of EqPPV DNA and antibodies

All DNA samples (n = 201) were screened for EqPPV using a quantitative probe-based real-time PCR targeting the myristylated protein gene (ORF093; bp 752–872, based on sequences obtained by next-generation sequencing), as previously described [11]. Samples with Ct values < 39 were considered positive (fluorescence threshold 0.003), whereas Ct values between 39 and 42 were considered borderline. To further confirm borderline samples (Ct > 39), an additional real-time PCR (SYBR Green as a fluorescence dye) was performed as previously described [11]. While the probe-based qPCR targets a short region of the EqPPV myristylated protein gene, the confirmatory PCR amplifies a longer, overlapping fragment (bp 349–872). To maximize the chance of detecting weak positive samples, a nested round was run with the same reaction conditions. All real-time PCRs were performed with AriaMx Real-time PCR System (Agilent Technologies). Previously confirmed EqPPV-positive and EqPPV-negative horse samples and water were used as controls for all assays. Nested PCR products were visualized in 1.5% agarose gel, and DNA fragment sizes were estimated using the GeneRuler 100 bp DNA Ladder (Thermo Fisher Scientific, Waltham, MA, USA). Borderline samples in probe-based PCR were only considered positive if they were also positive in SYBR-green-based PCR.

Rodent heart tissue extracts in PBS were tested for EqPPV-specific antibodies using AffiniPure Goat Anti-Mouse IgG (H + L, 1:100 dilution) and an immunofluorescence assay (IFA) as described previously [12], together with known positive and negative samples. Heart extract was not diluted prior to testing.

### Data analysis and visualization

A map of Finland with environmental sample collection sites was created using R (version 4.5.0) [23]. The plot was generated using the ‘ggplot2’ package [24], and spatial data analysis and visualization were done by the ‘sf’ package [25]. The map of Finland was obtained using ‘geofi’ [26]. Data manipulation was done using the ‘dplyr’ package [27], and ‘ggspatial’ [28] was applied to add cartographic elements. A heatmap was created using R [23] and ‘ggplot2’ [24] as above to visualize positive results. The confidence intervals were calculated with the Wilson score method.

## Results

### Epidemic surveillance between April 2022 and May 2025

A total of 25 horses (from 20 stables) were tested for EqPPV between April 2022 and May 2025. During the early phase of surveillance (April 2022-January 2025), 14 of these horses were tested with EqPPV PCR, and no new cases were detected. From February 2025 onwards, pastern dermatitis reports from stable/horse owners and veterinarians increased, and PCR diagnostics confirmed EqPPV DNA in eight out of ten stables (9/11 horses) that submitted samples between February and May 2025 (Additional file 1). Based on the referral form filled in during diagnostics, all positive horses from the eight positive stables were trotters from stables located in Tampere (n = 5), Lahti (n = 1), and Ylivieska (n = 1) (Fig. 1, location of one of the stables unreported) sub-regions in southern and western Finland, although reports from stables and veterinarians indicated large-scale spread all over the country. Clinical signs reported in the referral form were consistent with the previous pastern dermatitis outbreak, including papular/pustular/vesicular lesions, excretion, swelling, and redness in the pastern area. Five of the stables reported also having had pastern dermatitis cases in 2021–2022 (some not confirmed in the laboratory) while the information was not reported by the remaining three.

### Overview of EqPPV in case stables

Six out of eight PCR-positive stables participated in environmental sampling (Table 1) during which more detailed information on the timeline, clinical signs, and preventative measures than that reported in the referral form was collected by personal communication with the stable personnel. In most cases, environmental sampling was conducted within one to two weeks after the onset of clinical signs. Stable D and E were resampled three months later.

**Fig. 2:**
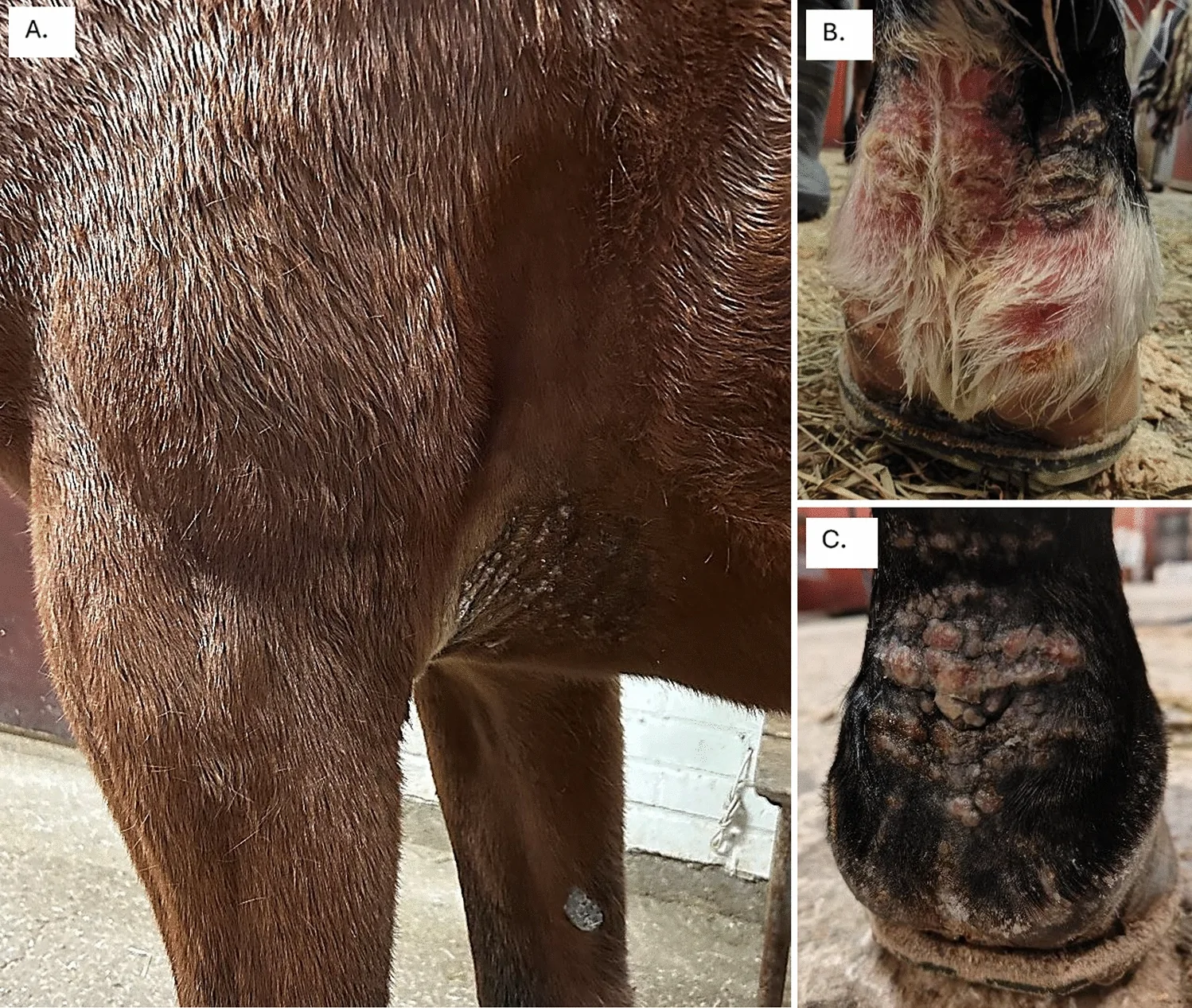
Axilla (**a**) and pastern (**b** and **c**) lesions of horses infected with EqPPV. **a** The horse (B_H1) was sampled two days after the onset of clinical signs. Lesions were also observed in the pastern area. The horse had undergone castration surgery one day prior to sampling. Equine parapoxvirus infection was confirmed by PCR, with Ct values of 36 from the armpit and 32 from the pastern. **b** and **c** are pasterns of affected horses from stable D

In stable A, clinical signs were observed in a horse (A_H1) imported from Sweden after the previous outbreak. Clinical signs in stable B were first detected in a horse (B_H1) that had undergone castration on 6 March 2025, but skin lesions had already appeared a few days earlier in the pastern area and near the left elbow (Fig. 2). In stable C, clinical signs first began in horse C_H1, which had recently moved from stable B to C.

Out of the six case stables, two reported only a single horse showing clinical signs of pastern dermatitis (A and F), while others had multiple (5–12) affected horses. Horses from stables A, D, and E had attended different race events, but no common event was identified as a shared source of infection. Control stables G and H had no reported clinical signs or PCR-positive findings.

Symptoms resembling the clinical signs seen in horses were reported in humans in contact with infected horses in 50.0% (3/6) of the case stables. A groom at stable A had a single circular, purple, papulovesiculopustular lesion of 1 cm diameter on the distal dorsal hand (sample A1, 10 days from symptom onset, Ct value 39.19). Lesion was already scabbed at the time of sampling, and the sample was borderline in PCR (Ct value 39.19). Stable D reported that two employees had had wounds on their hands that were exceptionally slow to heal, with recovery taking from January to April. One of these was PCR-positive (sample D1, Ct value 35.02). In stable E, one employee experienced a minor skin lesion on the wrist, but no sample was acquired. No human symptoms were reported in stables B, C, or F.

### Overview of preventative measures in case stables

Preventative measures differed between stables, mainly in terms of separation or isolation of affected horses and the level of hygiene and disinfection practices applied (Table 3). Although individual equipment and protective gloves were frequently used, isolation of affected horses was rarely possible.

**Table 3:** Preventative measures in case stables

Stable	Isolation of affected horses	Equipment use	Protective measures	Cleaning and disinfection	Additional notes
A	Yes	Individual equipment	Not reported	Not reported	
B	No	Bandages and boots washed before reuse	Gloves used when handling affected horses	Not reported	
C	No	Bandages and boots washed before reuse	Gloves used when handling affected horses	Not reported	
D	No	Equipment rinsed between horses	Not reported	Rinsing only	Several horses underperformed during outbreak
E	No	Individual equipment	Improved hand hygiene	Not reported	
F	No (horses together in the paddock)	Individual equipment	Gloves used	Betaine-based disinfectant. Affected horse’s box cleaned last	

### EqPPV DNA detection in environmental samples

**Fig. 3:**
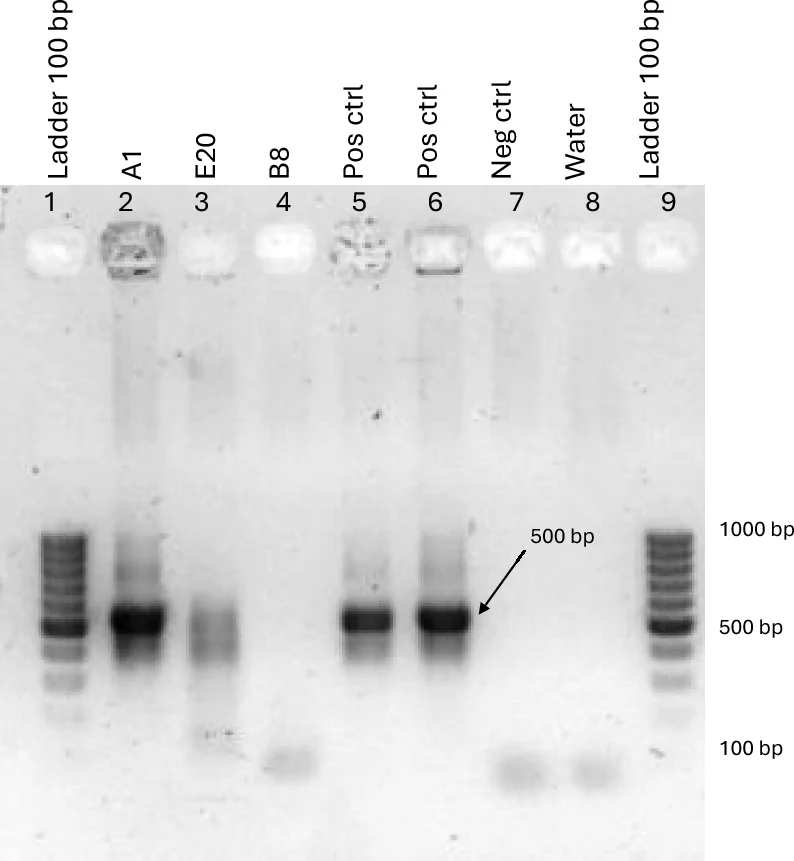
Agarose gel electrophoresis (1.5%) of the confirmatory real-time PCR products (ORF093; bp 349–872). To confirm borderline samples detected by probe-based EqPPV qPCR (Ct > 39), samples A1 (Ct 39.19) and E20 (Ct 39.11) were subjected to a confirmatory PCR **(**ORF093; bp 349–872**)**. Samples A1 and E20 showed a distinct band at approximately 500 bp. Both A1 and E20 were considered positive. Sample B8 (Ct 41.16) showed no specific amplification and was therefore considered negative

In the confirmatory nested PCR of borderline samples, samples A1 (probe-based EqPPV-PCR Ct 39.19) and E20 (probe-based EqPPV-PCR Ct 39.11) showed a distinct band at approximately 500 bp and were considered positive, whereas sample B8 (EqPPV-PCR Ct 41.16) was negative (Fig. 3).

Positive environmental samples were detected in 83.3% (5/6) of the case stables whereas no positive samples were detected in the control stables. Out of 138 environmental samples collected across six case stables, 17.4% (24/138) tested positive for EqPPV DNA. Results divided by environmental category and stable are presented in Table 2 and proportion/prevalence calculations are provided in Table 4. Comprehensive data are presented in Additional Files 2 and 3.

**Table 4:** Prevalence of EqPPV DNA in case stables according to environmental sample category and stable

	Proportion/Prevalence^a^	95% Cl
All samples	17.4% (24/138)	12.0–24.6
Category 1^b^	14.7% (5/34)	6.5–30.1
Category 2^b^	20.8% (11/53)	12.0–33.5
Category 3^b^	22.2% (4/18)	9.0–45.2
Category 4^b^	12.1% (4/33)	4.8–27.3
Stable A	5.0% (2/40)	1.4–16.5
Stable B	22.2% (2/9)	6.3–54.7
Stable C	0.0% (0/6)	0.0–39.0
Stable D	42.9% (12/28)	26.5–60.9
Stable E	10.0% (5/50)	4.4–21.4
Stable F	60.0% (3/5)	23.1–88.2

^a^Results are reported as percentages. Numbers (n/N) are shown in parentheses, and 95% confidence intervals (Wilson) are shown in second column

^b^Categories: human contact points (1), animal contact points (2), shared equipment/places (3), and environmental surfaces (4)

**Fig. 4:**
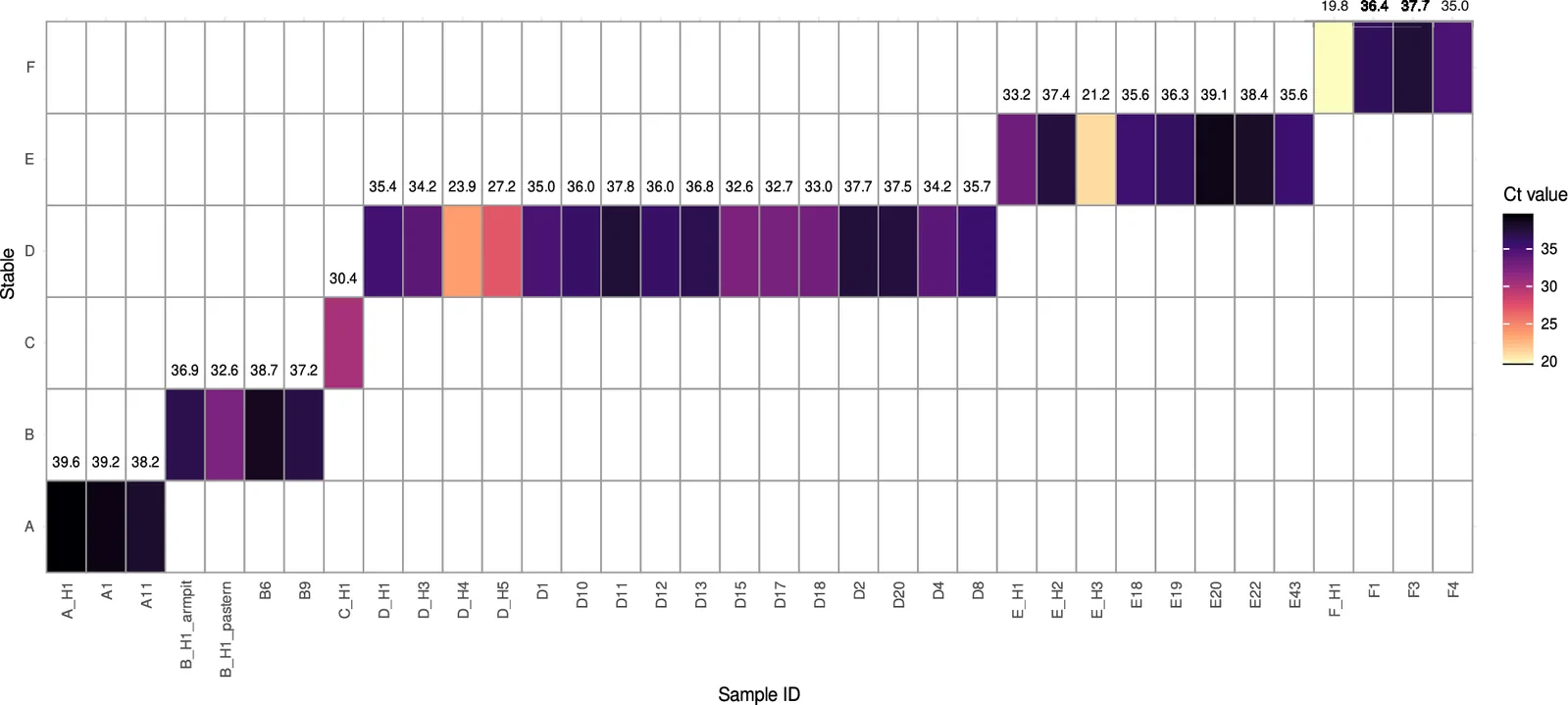
Heatmap of EqPPV PCR analyses of horse and environmental samples collected from the case stables. Stable A was sampled when the horse was almost healed. Stable C did not have PCR-positive environmental samples. Horse samples are marked with H and subsequent number. Dry swab samples (stables C, D, and F) were eluted into a smaller volume than Copan swab samples (stables A, B, and E). Due to this and the sampling not being carried out in a standardized way, direct quantitative comparison between stables and surface types should be avoided. Lower Ct values (yellow) indicate higher viral DNA concentration, suggesting active or recent infection. Higher Ct values (black) suggest lower viral DNA presence. The figure was generated using R (version 4.5.0)

EqPPV PCR-positive samples included several caretaker- and sampler-associated contact points: a scab on the caretaker’s hand on day 10 (healing phase), a wound on the owner’s hand, and the sampler’s glove. Among horse-associated items, positive findings were recorded from a bandage pad used on an affected horse, bedding of PCR-positive horses (B_H1, E_H1, and E_H3), and the inside of a bandage sampled weeks after use. Environmental surfaces that tested positive included the bottom of a sampler’s shoe (after sampling), a door handle in the tack room, the box door (horse F_H1), the wall between boxes adjacent to an affected horse, a drinking bowl, a blanket, a knee pad, the drain at the wash box, scissors, clippers, stable aisle robes, the wash box faucet, a broom, the wash box wall, and field shelter walls (Fig. 4).

### EqPPV in small mammals and bird samples from the racetrack

All faecal and urine samples from birds, rodents, and rabbits collected around the racetrack were negative for EqPPV DNA. Additionally, rodents tested negative for EqPPV antibodies.

## Discussion

Here, we followed the equine parapoxvirus situation in Finland for three years by testing samples from horses with clinical signs of pastern dermatitis, reported a new outbreak, and showed frequent presence of the virus DNA in environmental samples collected from affected stables. Our findings provide information on EqPPV spread and environmental presence, shedding light on its transmission within stables. These results underscore the importance of early detection of new outbreaks and implementing and maintaining routine preventative measures.

Based on serological data and reports from stable staff and veterinarians, EqPPV has been suspected to cause recurring wintertime outbreaks of pastern dermatitis in Finland every few years [11, 12]. In addition to the large-scale outbreak in 2021–2022, a serological study detected a peak in seroprevalence in 2015, although limited sample size prevented confirmation [12]. Following the 2021–2022 outbreak, no EqPPV cases were detected until early 2025, despite active testing of suspected pastern dermatitis cases. This pattern supports earlier predictions, although reasons behind the timing of outbreaks remain unclear. Group-immunity is a potential explanation, even though seroprevalence data among the horse population do not fully support it [12]. Similarly, asymptomatic carriers and low-scale circulation among horses outside outbreaks also cannot be excluded. For example, subclinical infections with RDPV have been reported [29]. While EqPPV has typically affected only the pastern area, one horse that developed lesions in the axillary region showed that EqPPV should be suspected also when typical signs are located in other parts of the body.

Our results on environmental samples align with similar studies on other PPVs like ORFV and BPSV. For example, ORFV DNA was successfully extracted from a water tank, scissors, and an ointment box [18, 20]. Similarly, several environmental samples tested positive for EqPPV DNA, highlighting possible indirect transmission routes within stables. DNA findings in shared tools, such as clippers, indicate that use of the same equipment for multiple horses may facilitate the spread, particularly when clipping horses causes small cuts or scratches on the skin. Three bedding samples that tested positive indicate a possible indirect transmission risk during cleaning through direct contact or airborne particles that may be released when the bedding is disturbed or fluffed up. A PCR-positive sample from a healthy horse’s box wall, adjacent to a horse with pastern dermatitis may reflect direct contact through the divider wall or transfer of contaminated bedding. DNA findings in wash boxes should be kept in mind not only in stables but also in racing events where they are used by multiple horses from different stables. The potential presence of EqPPV on surfaces combined with even minor skin injuries or abrasions sustained during races, could facilitate viral entry and increase infection risk. Although this study did not include environmental sampling of racetracks, future studies should address this gap.

EqPPV DNA was detected on a door handle, bottom of shoes (after sampling), stall door, wash box faucet, stable aisle robes, and floor broom, raising concern about humans spreading EqPPV within and potentially even between stables. This suggests that although EqPPV DNA was detected in several stables it may reflect indirect shedding or persistence of viral material rather than an active source of infection. Notably, stable C was the only stable that did not have positive environmental samples despite housing a EqPPV PCR-positive horse. This might be due to the uneven sampling intensity. Stable-level prevalence estimates should be interpreted with caution, as they are not directly comparable without adjustment for differing denominators. In particular, the absence of environmental positives in stable C despite the presence of a PCR-positive horse is likely attributable to limited environmental sampling rather than a true lack of environmental contamination with EqPPV DNA.

We have previously reported that symptoms in humans, including erythematous, vesicular, oedematous, exudative, scabby, and ulcerative skin lesions, were more commonly associated with stables experiencing EqPPV outbreaks, suggesting a zoonotic risk [11]. In the present study, similar symptoms were observed among stable personnel in three stables, with two PCR-positive cases. Interestingly, three stables that reported usage of gloves when handling infected horses reported no symptoms in humans. Although there is a possibility that the PCR-positive signal reflects environmental contamination with EqPPV DNA rather than true human infection, these findings support the possibility of EqPPV being zoonotic. This, however, remains unproven and requires targeted clinical sampling, serology, and sequencing during future outbreaks. While rare, potential spillover events have been documented, including human cases with poxvirus-like lesions following contact with equids [3].

ORFV has been shown to remain infectious in soil for at least six months under winter conditions and to persist in the host for six to eight weeks after infection initiation [17, 30]. Here, we identified EqPPV DNA in affected horses that were resampled weeks apart, which may indicate a potential for similar persistence of EqPPV; horse A_H1 was resampled one month apart (Ct values 31.00 and 39.64), and horse D_H5 was resampled two weeks apart (Ct values 27.22 and 28.18). Scabs containing the virus could fall off near the hooves and contaminate the surrounding environment such as track surface, bedding, or manure. A leg bandage sample (D2) remained PCR-positive even weeks after being outdoors, indicating some degree of persistence of EqPPV DNA. Since EqPPV has not yet been successfully propagated [4, 11, 30], the environmental persistence of viable virus remains uncertain. However, since other PPVs are known to persist in the environment [18, 19], EqPPV PCR-positive findings should be regarded as a potential infection risk to avoid virus spread, pending viability studies.

Small mammals are known reservoirs of some of the other poxviruses like cowpox virus [31]. All stool and urine samples of small mammals and heart extracts from rodents tested negative for EqPPV DNA or antibodies yielding no evidence of their potential role in EqPPV transmission. However, very limited sample size and scope render this analysis exploratory and the possibility of small mammals being reservoirs for EqPPV cannot be excluded. Arthropods such as ticks and flies, are known carriers of PPV DNA on animal farms [32, 33], therefore being possible mechanical vectors. Horses are frequently exposed to ticks in grassy paddocks and pastures and to flies. However, the EqPPV outbreaks in Finland have occurred during winter, when arthropod activity is minimal, suggesting these vectors likely played a limited or no role in EqPPV outbreak. These findings highlight the need to explore alternative transmission pathways.

Limitations of this study include the difficulty in sampling exact viral hotspots within the stable environment. For example, bedding is frequently changed and difficult to target, which may pose challenges to accurately assess the full extent of EqPPV spread. Also, PCR has been validated for equine samples, and assay performance in environmental matrices may be affected by inhibitors. As a result, sensitivity may vary depending on sample material, and negative results should be interpreted with caution, as false negatives may occur. The decision to choose a riding stable as a pure control was due to EqPPV circulation being more likely in trotting stables, whereas no PCR-confirmed cases have been reported in riding stables. Nonetheless, prior exposure cannot be entirely excluded, and differences in horse movement, management, and environmental conditions may introduce potential bias. Ct values should also not be considered quantitative. Despite all of this, EqPPV DNA was frequently detected on bedding and surfaces. Another limitation is that case numbers were partly based on stable reports rather than PCR confirmation, which may cause uncertainties. However, EqPPV was PCR-confirmed in all case stables. In the absence of a virus isolation method for EqPPV [4, 11, 30], we cannot conclude that PCR-confirmed EqPPV in environmental samples was infective.

We recommend the following practical preventative measures to help reduce the spread of EqPPV in equine facilities, as shared stable environments can contribute to indirect virus transmission. While not all practices may be achievable in every stable, implementing as many measures as possible can help reduce disease transmission. Based on our findings here and in earlier studies, affected horses should be isolated, and separate or cleaned and disinfected equipment should be used for their care. Infected horses should be treated, and their boxes should be cleaned and disinfected last, minimizing the risk of spreading pathogens via hands, clothing, or footwear. After handling horses with pastern dermatitis, hands should be washed and disinfected, while shoes should be disinfected or one should consider using disposable gloves and shoe covers. Surfaces should be cleaned and disinfected using effective agents. Additionally, shared tools, such as clippers, should be cleaned and disinfected between horses to prevent cross-contamination. Contaminated bedding should be removed and discarded, followed by cleaning and disinfection. Until experimental confirmation of the effect of different disinfectants on EqPPV is acquired, we recommend using disinfectants proven to be effective against other poxviruses. For example, 5% sodium hypochlorite or Virkon®, has been recommended for poxviruses (World Organization for Animal Health) [34]. The legs of horses should be frequently checked for signs of the disease during winters and outbreaks, and regular testing of the racetrack environment should be considered to enable early recognition and control measures. If applied consistently, these measures could play a key role in limiting the spread of the virus in the stable environment.

## Conclusions

This study confirms a recent EqPPV outbreak in trotting stables and demonstrates the presence of EqPPV DNA in clinically affected horses and the stable environment. Detection of EqPPV DNA on common equipment and spaces as well as on personnel shows a widespread environmental distribution during the outbreak. Although PCR findings do not confirm infectivity, these results and the known environmental persistence of other PPVs raise the possibility that environmental presence may contribute to transmission dynamics within the affected stables. The significance of these indirect transmission routes requires more studies assessing virus viability and effectiveness of different preventative protocols. Overall, these findings underscore the high potential for reoccurring outbreaks and the importance of continued surveillance and early implementation of hygiene and preventative practices to minimize the effect of future outbreaks.

## Supplementary Information

Additional file 1: A Flowchart of equine parapoxvirus (EqPPV) diagnostics and environmental sampling. Diagnostic testing for EqPPV was conducted between April 2022 and May 2025 in 20 stables (n = 25 horses). Between April 2022 and January 2025, all horses tested negative for EqPPV DNA (n = 14). During the outbreak (February-May 2025), EqPPV DNA was detected in eight out of ten stables (9/11 horses). Environmental sampling (n=161) was performed in six of the eight stables with PCR-confirmed EqPPV. Case stables (6/8) contributed 138 samples, of which 24 were EqPPV DNA-positive, while control stables (2/8) provided 23 samples, all of which were negative. Positive environmental samples are further divided into four sampling categories. Categories: human contact points (1), animal contact points (2), shared equipment/places (3), and environmental surfaces (4).

Additional file 2: Information on environmental sampling of case and control stables.

Additional file 3: Information on horse samples from stables that participated in environmental sampling.

Additional file 4: Information on small mammals and bird samples collected from the racetracks in 2024.

## Data Availability

The datasets supporting the conclusions of this article are included within the article and its additional files. Data that could potentially be used to identify participants are excluded for privacy reasons.

## Abbreviations

BPSV: Bovine popular stomatitis virus
EqPPV: Equine parapoxvirus
GSEPV: Seal parapoxvirus
IFA: Immunofluorescence assay
ORFV: Orf virus
PBS: Phosphate-buffered saline
PCPV: Pseudocowpox virus
PPV: Parapoxvirus
RDPV: Parapoxvirus red deer
RT: Room temperature
WOAH: World Organization for Animal Health
