# Supplementary material for: Detection of equine parapoxvirus in the stable environment during pastern dermatitis outbreak in Finland
Source: Acta Vet Scand. 2026 Jun 30;68:39. doi: 10.1186/s13028-026-00870-9 (PMC13613859; doi:10.1186/s13028-026-00870-9)
Supplement: Supplementary file 1 [file 13028_2026_870_MOESM1_ESM.pdf]

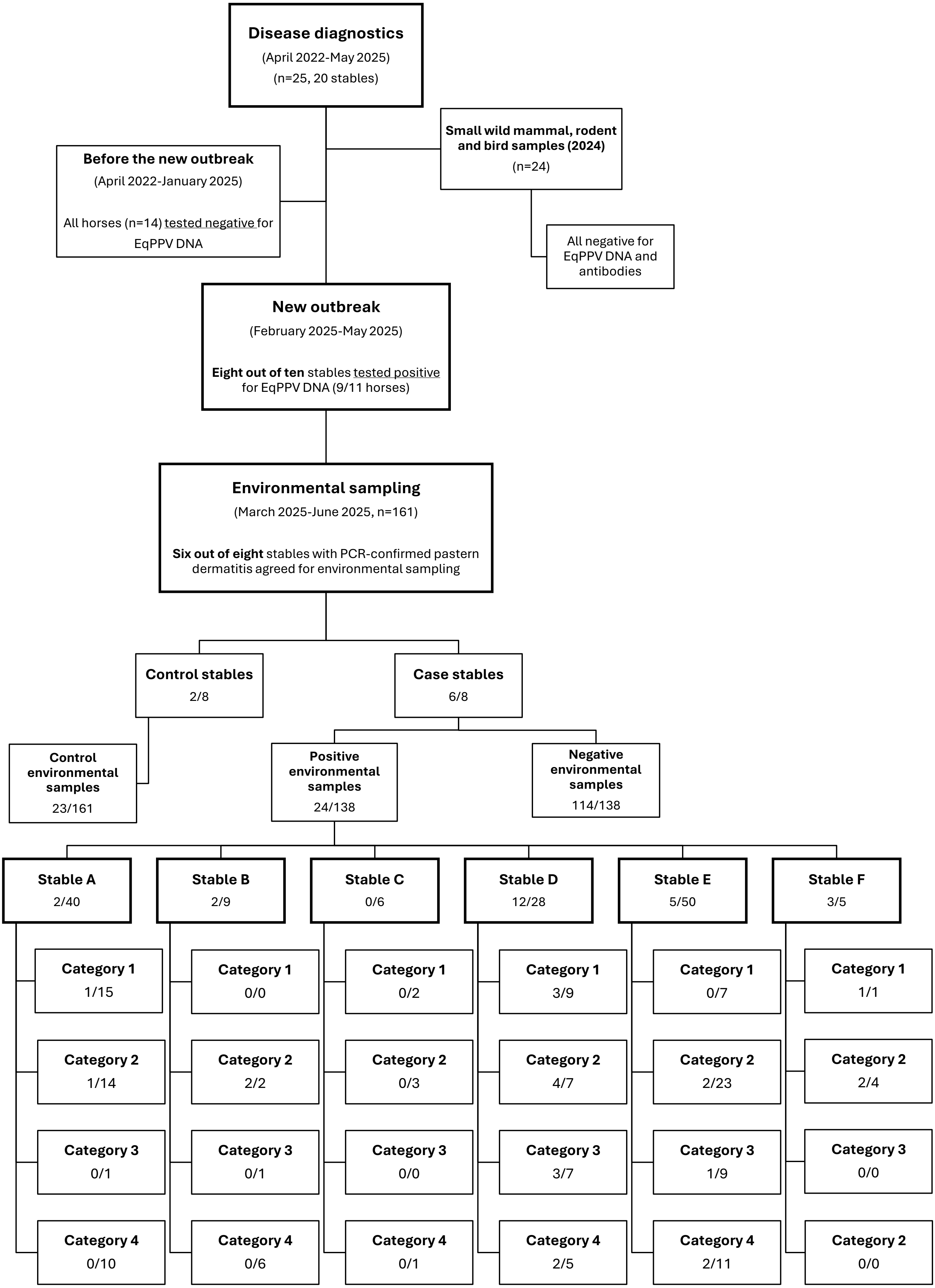

Disease diagnostics
(April 2022-May 2025)
(n=25, 20 stables)
Before the new outbreak
(April 2022-January 2025)
All horses (n=14) tested negative for EqPPV DNA
Small wild mammal, rodent and bird samples (2024)
(n=24)
All negative for EqPPV DNA and antibodies
New outbreak
(February 2025-May 2025)
Eight out of ten stables tested positive for EqPPV DNA (9/11 horses)
Environmental sampling
(March 2025-June 2025, n=161)
Six out of eight stables with PCR-confirmed pastern dermatitis agreed for environmental sampling
Control stables
2/8
Case stables
6/8
Control environmental samples
23/161
Positive environmental samples
24/138
Negative environmental samples
114/138
Stable A
2/40
Category 1
1/15
Category 2
1/14
Category 3
0/1
Category 4
0/10
Stable B
2/9
Category 1
0/0
Category 2
2/2
Category 3
0/1
Category 4
0/6
Stable C
0/6
Category 1
0/2
Category 2
0/3
Category 3
0/0
Category 4
0/1
Stable D
12/28
Category 1
3/9
Category 2
4/7
Category 3
3/7
Category 4
2/5
Stable E
5/50
Category 1
0/7
Category 2
2/23
Category 3
1/9
Category 4
2/11
Stable F
3/5
Category 1
1/1
Category 2
2/4
Category 3
0/0
Category 2
0/0
